# Efficacy of a modified FRAIL scale in predicting the peri-operative outcome of hepatectomy in older adults (aged ≥ 75 years): a model development study

**DOI:** 10.1186/s12877-023-04488-8

**Published:** 2023-11-23

**Authors:** Lining Xu, Weiyu Wang, Yingying Xu, Bo Yang

**Affiliations:** 1 Department of General Surgery, The Second Medical Center & National Clinical Research Center for Geriatric Diseases, Chinese PLA General Hospital, Beijing, 100853 China; 2 Zhongnan Hospital of Wuhan University, Institute of Hepatobiliary Diseases of Wuhan University, Transplant Center of Wuhan University, National Quality Control Center for Donated Organ Procurement, Hubei Key Laboratory of Medical Technology on Transplantation, Wuhan, 430071 China; 3 Department of Internal Medicine, Henan Cancer Hospital, Zhengzhou, 450003 China; 4 Department of Radiology, Affiliated Union Hospital, Tongji Medical College, Huazhong University of Science and Technology, Wuhan, 430022 China

**Keywords:** Frailty, Hepatectomy, Older adults, Risk assessment tool

## Abstract

**Background:**

The FRAIL scale for evaluating frailty consists of five items: fatigue, resistance, aerobic, illness, and loss of weight. However, it is difficult to obtain a specific weight loss value. Since the Timed Up and Go Test (TUGT) is simple, accurate, and easy to perform, we replaced weight loss with the TUGT in the FRAIL scale, with the remaining four items unchanged, and named it the FRAIT scale. The aim of this study was to determine the value of the FRAIT scale in predicting the peri-operative outcome of hepatectomy.

**Methods:**

This model development study was conducted between January 2017 and December 2021. The reliability, validity and area under the curve (AUC) of the FRAIL/FRAIT scales were calculated. The frailty status of patients aged ≥ 75 years who underwent hepatectomy was measured using the FRAIL/FRAIT scales. Logistic regression was used to compare the relationship between FRAIL/FRAIT scores/grades and perioperative outcomes.

**Results:**

The AUCs for predicting operation duration, intraoperative bleeding, complications, and death based on the FRAIL score were 0.692, 0.740, 0.709, and 0.733, respectively, and those based on the FRAIT score were 0.700, 0.745, 0.708, and 0.724, respectively. The AUCs for predicting operation duration, intraoperative bleeding, complications, and death based on the FRAIL grade were 0.693, 0.735, 0.695, and 0.755, respectively, and those based on the FRAIT grades were 0.700, 0.758, 0.699, and 0.750, respectively. The FRAIL score has three effective predictors (intraoperative bleeding, complications, and death), while the FRAIT score has four effective predictors (operation duration, intraoperative bleeding, complications, and death). The FRAIL grade has two effective predictors (intraoperative bleeding and death), while the FRAIT grade has three effective predictors (operation duration, intraoperative bleeding, and death).

**Conclusions:**

This study describes a new and more effective tool for the assessment of preoperative frailty in older adults undergoing hepatectomy. The items of the FRAIT scale are easier to obtain than those of the FRAIL scale, and the predictive effect of the FRAIT scale is stronger than that of the FRAIL scale.

## Background

Owing to the particularities of older patients, the requirements for surgical safety are significantly higher than those of other age groups [1]. Therefore, it is necessary to adopt high-quality preoperative evaluation strategies for older patients to meet the growing demand for surgery and to ensure safety. Frailty is an independent predictor of a high incidence of postoperative adverse events [2–4]. Frailty symptoms in older patients should be evaluated, frailty scores should be recorded before surgery, and geriatricians should be consulted for further evaluation if necessary [5]. The FRAIL Screening Scale (FRAIL scale) is a relatively simple and suitable tool for rapid clinical evaluation [6], and is commonly used for frailty evaluation in the clinic [7]. The FRAIL scale contains five items: [8] fatigue, resistance, aerobic, illness, and loss of weight (exceeding 5% in the previous year). However, the FRAIL scale has a limitation, which is weight loss. It is difficult to assess weight loss because most people do not measure their weight daily, making it difficult to determine whether their weight loss has reached 5%. Moreover, most hospitalized patients have not measured their weight within 1 year before admission; hence, it is impossible to measure weight loss in the preoperative evaluation of patients, which limits the application of the FRAIL scale in preoperative evaluation. Therefore, we recommend the use of a precise and easy-to-evaluate item instead of weight loss.

The Timed Up and Go Test (TUGT) combines various actions that easily lead to falls, such as standing up, sitting down, walking, and turning [9]. Since falls are closely related to functional and physical status and the TUGT is simple, accurate, and easy to perform, we replaced weight loss with TUGT as a means of preoperative assessment of frailty [10]. The remaining four items remained unchanged; that is, the FRAIL scale was changed to the FRAIT scale. Liver surgery is a difficult, high-risk procedure [11, 12], and the perioperative outcomes of hepatectomy, such as long operation duration, intraoperative blood loss, postoperative complications, death, and prolonged hospital stay, are related to frailty status.

Therefore, the aim of this study was to compare the role of the FRAIT and FRAIL scales in predicting peri-operative outcomes after hepatectomy and to determine the value of the FRAIT scale in predicting the peri-operative outcome of hepatectomy.

In addition, we tested the modified version of the FRAIL scale, in which the weight loss item has been removed (containing the remaining four items, named the Reduced – FRAIL scale), verified its reliability and validity, and evaluated its value in predicting frailty.

## Methods

### Study design

This study included patients (aged ≥ 75 years) undergoing hepatectomy at 4 hospitals between January 2017 and December 2021. All institutions obtained their respective approvals according to their local hospital’s requirements. These were collated and analyzed centrally at the Chinese PLA General Hospital.

### Setting and participants

**Fig. 1:**
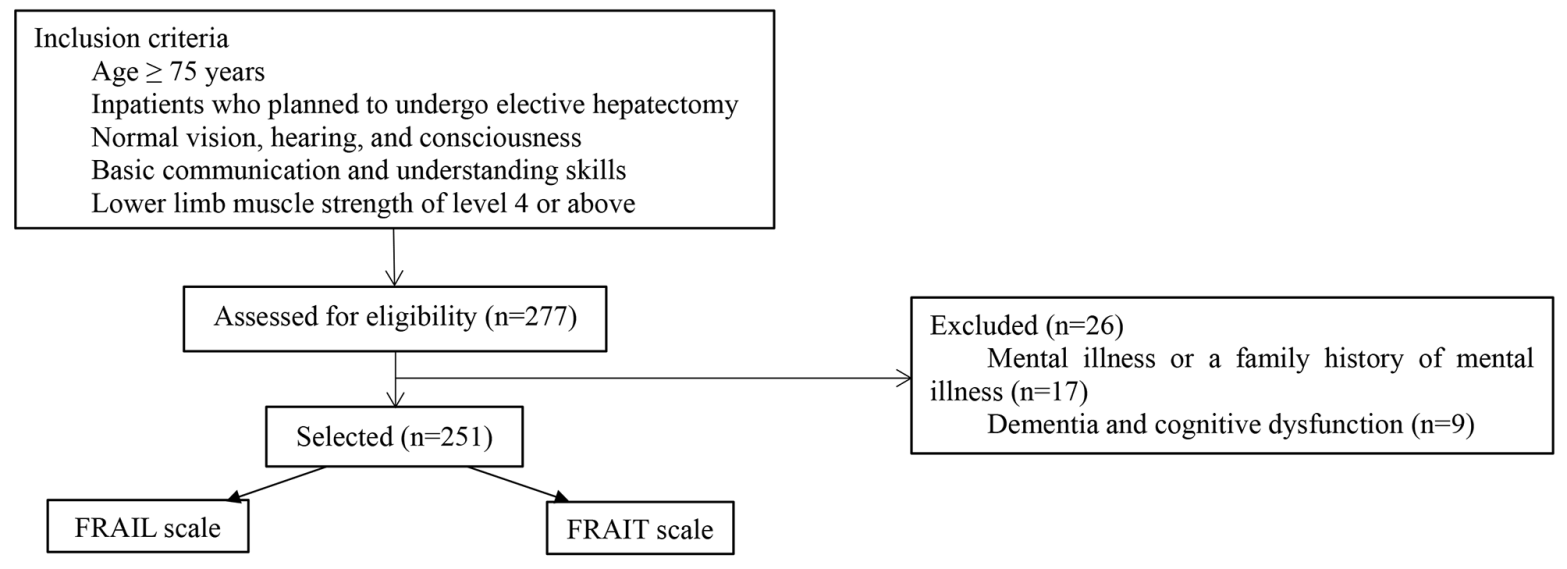
Flow chart of patients selection

This study was performed in 4 hospitals (located in Beijing, Zhengzhou, and Wuhan) in China. The inclusion criteria were as follows: (1) age ≥ 75 years; (2) planned to undergo elective hepatectomy; (3) normal vision, hearing, and consciousness; (4) basic communication and understanding skills; and (5) lower limb muscle strength of level 4 or above, that is, the patient could independently complete the movement from sitting to standing and walking. The exclusion criteria were as follows: (1) mental illness or a family history of mental illness and (2) dementia and cognitive dysfunction (Fig. 1).

All study participants had clear surgical indications, and the first choice of treatment was surgery; therefore, no neoadjuvant treatment was administered before the procedure. Malignant and benign lesions indicated for hepatectomy in elderly patients mainly included hepatocellular carcinoma, hepatolithiasis and hepatic hemangiomas. For malignant lesions indicated for specific surgeries, refer to the “Chinese guidelines for the diagnosis and treatment of primary liver cancer [13]. In China, hepatolithiasis is the most common benign liver disease that requires surgery. Patients with hepatolithiasis often develop infection and abnormal liver function because of their special pathological characteristics. These complications can lead to local liver damage, and partial hepatectomy is needed in these cases. Regardless of whether the disease is benign or malignant, it is necessary to accurately evaluate liver reserve function before surgery. Patients with insufficient liver reserve function to maintain normal liver metabolism after surgery are not suitable for surgery. In this study, the “individualized evaluation and decision-making system for the safety limit of hepatectomy” proposed in the Chinese “consensus on evaluation of hepatic functional reserve before hepatectomy” was used to evaluate liver reserve function [14]. Only those with surgical indications underwent surgery.

Before the investigation, the examiner explained the content of the scale and trained the patients to perform the TUGT test [15]. The examiner collected demographic and sociological data, health data, and other relevant information about the patient.

### Definitions

TUGT ≥ 15 s indicates that the patient is frail, and is calculated by 1 point. The modified FRAIL scale - FRAIT Scale, is shown in Table 1. To assess fraility using the FRAIT scale, refer to the FRAIL scale criteria for assessing frailty; that is, the frailty score ranges 0‒5 points. Health status was divided into three grades: frail (3‒5 points), prefrail (1‒2 points), and strong (0 points).

**Table 1:** FRAIT scale

Items	Questions	Scores
Fatigue	Fatigue most or all of the time in the past 4 weeks	Yes(1 point) No(0 point)
Resistance	Difficulty climbing up the stairs without taking a break, using auxiliary tools, or help from others	Yes(1 point) No(0 point)
Aerobic	Difficulty walking a block (500 m) without using auxiliary tools or help from others	Yes(1 point) No(0 point)
Illness	Suffering from more than 5 diseases^#^	Yes(1 point) No(0 point)
TUGT	≥ 15s	Yes(1 point) No(0 point)

^#^ hypertension, diabetes, cancer (other than a minor skin cancer), chronic lung disease, heart attack, congestive heart failure, angina, asthma, arthritis, stroke, and kidney disease

The reliability and validity of the FRAIL and FRAIT scales were compared according to the frailty score and frailty grade. Logistic regression was used to analyze the relationship between the FRAIL/FRAIT scale scores/grades and the factors related to the perioperative outcomes of hepatectomy. This helped to clarify the relationship between the patients’ frailty status assessed by the FRAIL/FRAIT scales and their peri-operative outcomes to reveal the value of the FRAIL/FRAIT scales in predicting the peri-operative outcomes of hepatectomy to compare their predictive strength.

The relevant factors of perioperative outcomes included operation duration, intraoperative blood loss, postoperative complications, death, and postoperative hospital stay.

### Statistical analysis

Descriptive statistics were reported as the mean ± standard deviation or percentage. Cronbach’s α coefficient was used to evaluate the reliability of the scale, and a coefficient > 0.7 indicated that the scale had good reliability. The Kaiser–Meyer–Olkin (KMO) coefficient was used to evaluate the validity of the scale, and a coefficient > 0.6 indicates that the scale has good validity. Univariate analysis was used to compare the FRAIL scale and the new FRAIT scale status (frail, prefrail, and strong) of the frail population characteristics. Bivariate logistic regression was used to investigate the correlations between FRAIL/FRAIT scale scores/grades and factors related to peri-operative outcomes. Logistic regression analysis was used to report adjusted odds ratios, Wald, and *P* values.

According to the FRAIL/FRAIT scale scores/grades of hepatectomy in older adults, the receiver operating characteristic (ROC) curve was used to calculate the predicted area under curve (AUC) of each peri-operative outcome-related factor to determine the relationship between the scores/grades of the FRAIL/FRAIT scales of patients who underwent hepatectomy and the relevant factors of peri-operative outcomes related to hepatectomy. This helped to further evaluate and compare the value of the new FRAIT scale and the existing FRAIL scale in predicting the peri-operative outcomes of hepatectomy.

## Results

A total of 251 cases were identified from January 2017 to December 2021. Using the FRAIL scale, 41 patients (16.33%) were frail, 100 (39.84%) were prefrail, and 110 (43.82%) were strong. Patient baseline characteristics and further details are provided in Table 2.

**Table 2:** The patient baseline characteristics

Factors	*n*	FRAIL scale			
		Strong	Prefrail	Frail	*P*
General background					
Sex					
Female	85	41(48.23%)	31(36.47%)	13(15.29%)	0.396
Male	166	69(41.57%)	69(41.57%)	28(16.88%)	
Comorbidity					
Yes	42	1(2.38%)	9(21.43%)	32(76.19%)	< 0.001
No	209	109(52.15%)	91(43.54%)	9(4.31%)	
Diagnosis					
Malignant diseases	193	83(43.01%)	79(40.93%)	31(16.06%)	0.733
Benign diseases	58	27(46.55%)	21(36.21%)	10(17.24%)	
Preoperative evaluation					
Blood test					
Albumin (g/L)					
< 35	62	9(14.52%)	20(32.26%)	33(53.23%)	< 0.001
≥ 35	189	101(53.44%)	80(42.33%)	8(4.23%)	
T-BIL^#^ (µmol/L)					
≤ 21	188	108(57.45%)	66(35.11%)	14(7.45%)	< 0.001
> 21	63	2(3.17%)	34(53.97%)	27(42.86%)	
Child-pugh					
A	214	108(50.47%)	95(44.39%)	11(5.14%)	< 0.001
B/C	37	2(5.41%)	5(13.51%)	30(81.08%)	
Tumor-related factors					
Largest tumor size (cm)					
≤ 5	134	59(44.03%)	48(35.82%)	27(20.15%)	0.156
> 5	117	41(35.04%)	37(31.62%)	39(33.33%)	
Number of lesions					
Single	218	94(43.12%)	90(41.28%)	34(15.60%)	0.308
Multiple	33	16(48.48%)	10(30.30%)	7(21.21%)	
Operative variables					
Resection scope					
Minor hepatectomy	175	81(46.29%)	68(38.86%)	26(14.86%)	0.428
Major hepatectomy	76	29(38.16%)	32(42.11%)	15(19.74%)	
Resection style					
Nonanatomical	172	74(43.02%)	69(40.11%)	29(16.86%)	0.914
Anatomical	79	36(45.57%)	31(39.24%)	12(15.19%)	
Operative duration (min)					
< 180	126	77(61.11%)	39(30.95%)	10(7.94%)	< 0.001
≥ 180	125	33(26.40%)	61(48.80%)	31(24.80%)	
Blood loss (mL)					
≤ 800	229	108(47.16%)	89(38.86%)	32(13.97%)	< 0.001
> 800	22	2(9.09%)	11(50.00%)	9(40.91%)	
Blood transfusion					
Yes	89	4(4.49%)	57(64.04%)	28(31.46%)	< 0.001
No	162	106(65.43%)	43(26.54%)	13(8.02%)	
Complication					
Yes	204	99(48.53%)	85(41.67%)	20(9.80%)	< 0.001
No	47	11(23.40%)	15(31.91%)	21(44.68%)	
Death					
Yes	5	0(0.00%)	3(60.00%)	2(40.00%)	0.106
No	246	110(44.72%)	97(39.43%)	39(15.85%)	
Preoperative stay (days)					
≤ 7	199	87(43.72%)	76(38.19%)	36(18.09%)	0.293
> 7	52	23(44.23%)	24(46.15%)	5(9.62%)	
Postoperative stay (days)					
≤ 14	174	87(50.00%)	67(38.51%)	20(11.49%)	0.001
> 14	77	23(29.87%)	33(42.86%)	21(27.27%)	

^#^ T-BIL: total bilirubin

According to the FRAIT scale, 44 patients (17.53%) were frail, 96 (38.25%) were prefrail, and 111 (44.22%) were strong. The patient-specific outcomes are presented in Table 3.

**Table 3:** The patient specific outcomes

Factors	FRAIT scale			
	Strong	Prefrail	Frail	*P*
General background				
Sex				
Female	42(49.41%)	31(36.47%)	12(14.12%)	0.189
Male	69(41.57%)	65(39.16%)	32(19.28%)	
Comorbidity				
Yes	1(2.38%)	11(26.19%)	30(71.43%)	< 0.001
No	110(52.63%)	85(40.67%)	14(6.70%)	
Diagnosis				
Malignant diseases	84(43.52%)	74(38.34%)	35(18.13%)	0.581
Benign diseases	27(46.55%)	22(37.93%)	9(15.52%)	
Preoperative evaluation				
Blood test				
Albumin (g/L)				
< 35	9(14.52%)	21(33.87%)	32(51.61%)	< 0.001
≥ 35	102(53.97%)	75(39.68%)	12(6.35%)	
T-BIL^#^ (µmol/L)				
≤ 21	109(57.98%)	66(35.11%)	13(6.91%)	< 0.001
> 21	2(3.17%)	30(47.62%)	31(49.21%)	
Child-pugh				
A	109(50.93%)	88(41.12%)	17(7.94%)	< 0.001
B/C	2(5.41%)	8(21.62%)	27(72.97%)	
Tumor-related factors				
Largest tumor size (cm)				
≤ 5	59(44.03%)	48(35.82%)	27(20.15%)	0.459
> 5	52(44.44%)	48(41.03%)	17(14.53%)	
Number of lesions				
Single	94(43.12%)	87(39.91%)	37(16.97%)	0.381
Multiple	17(51.52%)	9(27.27%)	7(21.21%)	
Operative variables				
Resection scope				
Minor hepatectomy	82(46.86%)	65(37.14%)	28(16.00%)	0.397
Major hepatectomy	29(38.16%)	31(40.79%)	16(21.05%)	
Resection style				
Nonanatomical	75(43.60%)	65(37.79%)	32(18.60%)	0.806
Anatomical	36(45.57%)	31(39.24%)	12(15.19%)	
Operative duration (min)				
< 180	78(61.90%)	38(30.16%)	10(7.94%)	< 0.001
≥ 180	33(26.40%)	58(46.40%)	34(27.20%)	
Blood loss (mL)				
≤ 800	109(47.60%)	87(37.99%)	33(14.41%)	< 0.001
> 800	2(9.09%)	9(40.91%)	11(50.00%)	
Blood transfusion				
Yes	107(65.64%)	46(28.22%)	10(6.13%)	< 0.001
No	4(4.55%)	50(56.82%)	34(38.63%)	
Complication				
Yes	11(23.40%)	14(29.79%)	22(46.81%)	< 0.001
No	100(49.02%)	82(40.20%)	22(10.78%)	
Death				
Yes	0(0.00%)	3(60.00%)	2(40.00%)	0.114
No	111(45.12%)	93(37.80%)	42(17.07%)	
Preoperative stay (days)				
≤ 7	87(43.72%)	75(37.69%)	37(18.59%)	0.689
> 7	24(46.15%)	21(40.38%)	7(13.46%)	
Postoperative stay (days)				
≤ 14	88(50.57%)	66(37.93%)	20(11.49%)	< 0.001
> 14	23(29.87%)	30(38.96%)	24(31.17%)	

^#^ T-BIL: total bilirubin

The reliability and validity of the FRAIL scale were also analyzed: the Cronbach’s α coefficient based on standardized items was 0.735, the KMO coefficient was 0.650, and the significance of Bartley’s spherical test was 0.000. The reliability and validity of the Reduced-FRAIL scale were analyzed: the Cronbach’s α coefficient based on standardized items was 0.597, the KMO coefficient was 0.565, and the significance of Bartley’s spherical test was 0.000. Furthermore, the reliability and validity of the FRAIT scale were analyzed: the Cronbach’s α coefficient based on standardized items was 0.737, the KMO coefficient was 0.652, and the significance of Bartley’s spherical test was 0.000.

### Criterion-related validity

The Pearson correlation coefficient between the FRAIT and FRAIL scales was 0.980, showing a strong correlation between them.

Logistic regression analysis was used to analyze the relationship between FRAIL/FRAIT scale scores (0‒5 points)/grades (strong, prefrail, and frail) of patients who underwent hepatectomy and factors related to the perioperative outcomes of hepatectomy (including operation duration, blood loss, complications, mortality, preoperative hospital stay, and postoperative hospital stay). It was also used to clarify the relationship between the patients’ frailty status assessed by the FRAIL/FRAIT scales and their surgical prognosis. Furthermore, it helped to reveal the value of the FRAIL/FRAIT scales in predicting the peri-operative outcomes of hepatectomy and to compare their predictive intensity. Further details are provided in Table 4.

**Table 4:** The relationship between FRAIL/FRALT scales and peri-operative outcomes

Variable	OR	Wald	*P*	OR	Wald	*P*
	FRAIL score			FRAIT score		
Operation duration	1.736	22.236	< 0.001	1.778	24.284	< 0.001
Blood loss	1.693	15.079	< 0.001	1.707	15.489	< 0.001
Complication	1.790	26.602	< 0.001	1.756	25.766	< 0.001
Mortality Preoperative hospital stay	1.549 0.882	2.937 1.039	0.087 0.308	1.439 0.890	1.945 0.929	0.163 0.335
Postperative hospital stay	1.505	16.546	< 0.001	1.535	18.448	< 0.001
	FRAIL grade			FRAIT grade		
Operation duration	2.931	28.889	< 0.001	3.023	31.155	< 0.001
Blood loss	3.364	13.919	< 0.001	3.912	16.661	< 0.001
Complication	2.982	21.172	< 0.001	2.957	21.568	< 0.001
Mortality Preoperative hospital stay	3.566 0.840	3.691 0.631	0.055 0.427	3.367 0.869	3.481 0.431	0.062 0.511
Postperative hospital stay	1.976	12.537	< 0.001	2.096	15.142	< 0.001

The AUC value was calculated using the ROC curve to evaluate the relationship between FRAIL/FRAIT scale scores/grades and peri-operative outcome-related factors (including operation duration, blood loss, complications, and death).

**Fig. 2:**
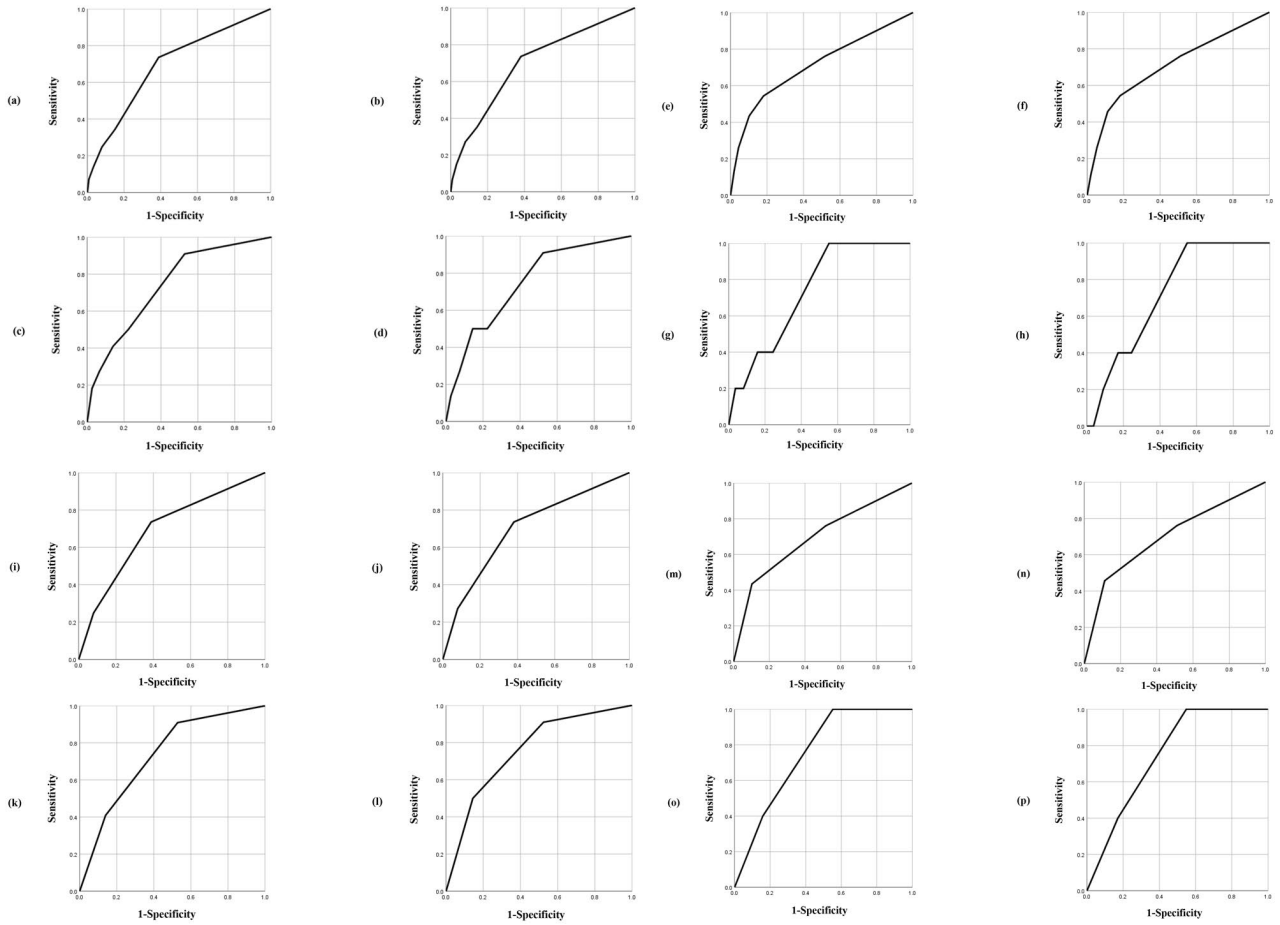
**The predictive values of FRAIL/FRAIT scales**. (**a**) The predictive value of the FRAIL score for operation duration: the area under the curve (AUC) was 0.692. (**b**) The predictive value of the FRAIT score for operation duration: the AUC was 0.700. (**c**) The predictive value of the FRAIL score for intraoperative bleeding: the AUC was 0.740. (**d**) The predictive value of the FRAIT score for intraoperative bleeding: the AUC was 0.745. (**e**) The predictive value of the FRAIL score for complications: the AUC was 0.709. (**f**) The predictive value of the FRAIT score for complications: the AUC was 0.708. (**g**) The predictive value of the FRAIL score for perioperative mortality: the AUC was 0.733. (**h**) The predictive value of the FRAIT score for perioperative mortality: the AUC was 0.724. (**i**) Prediction value of FRAIL grade for operation duration: AUC was 0.693. (**j**) Prediction value of FRAIT grade for operation duration: AUC was 0.700. (**k**) The predictive value of FRAIL grade for intraoperative bleeding: AUC was 0.735. (**l**) The predictive value of the FRAIT grade for intraoperative bleeding: the AUC was 0.758. (**m**) The predictive value of the FRAIL grade for complications: the AUC was 0.695. (**n**) Prediction value of the FRAIT grade for complications: the AUC was 0.699. (**o**) Prediction value of FRAIL grade for perioperative mortality: AUC was 0.755. (**p**) Prediction value of FRAIT grade for perioperative mortality: AUC was 0.750

The predictive value of the FRAIL and FRAIT scores for operation duration was 0.692 and 0.700, respectively. The predictive value of the FRAIL and FRAIT scores for intraoperative bleeding was 0.740 and 0.745, respectively. The predictive value of the FRAIL and FRAIT scores for complications was 0.709 and 0.708, respectively. The predictive value of the FRAIL and FRAIT scores for peri-operative mortality was 0.733 and 0.724, respectively. Further details are provided in Fig. 2.

The predictive values of the FRAIL and FRAIT grades for operation duration were 0.693 and 0.700, respectively. The predictive value of the FRAIL and FRAIT grades for intraoperative bleeding was 0.735 and 0.758, respectively. The predictive effect of the FRAIL and FRAIT grades for complications was 0.695 and 0.699, respectively. The predictive effect of the FRAIL and FRAIT grades for peri-operative mortality was 0.755 and 0.750, respectively. Further details are provided in Fig. 2.

The results showed that there were three effective predictors of the FRAIL score (intraoperative bleeding, complications, and death) and two effective predictors of the FRAIL grade (intraoperative bleeding and death) when predicting the relevant factors of peri-operative outcomes. The predictive effect of the FRAIL score was stronger than that of its grade. There were four effective predictors of the FRAIT score (operation duration, intraoperative bleeding, complications, and death) and three effective predictors of the FRAIT grade (operation duration, intraoperative bleeding, and death). The predictive effect of the FRAIT score was stronger than that of its grade.

In addition, the results indicated that when predicting the factors related to peri-operative outcomes, the FRAIL score has three effective predictors (intraoperative bleeding, complications, and death), and the FRAIT score has four effective predictors (operation duration, intraoperative bleeding, complications, and death). The predictive effect of the FRAIT score was stronger than that of the FRAIL score. The FRAIL grade had two effective predictors (intraoperative bleeding and death), and the FRAIT grade had three effective predictors (operation duration, intraoperative bleeding, and death). The predictive effect of the FRAIT grade was stronger than that of the FRAIL grade.

## Discussion

This study describes a new and more effective tool for the assessment of preoperative frailty in older adults undergoing hepatectomy. The items of the FRAIT scale are easier to assess than those of the FRAIL scale, and the predictive value of the FRAIT scale is better than that of the FRAIL scale.

In recent years, the number of surgeries performed on older patients has increased faster than the rate of aging, and the use of “frailty” in the preoperative risk assessment of older patients is of great interest [16, 17]. The concept of frailty originated in geriatrics [18]. Frailty refers to the status of accumulated decline in the functions of multiple systems, leading to a decline in the body’s reserve capacity and resistance ability [19]. This status increases the risk of death, disability, delirium, falls, long-term hospitalization, and other adverse events, reflecting heterogeneity in the health of older adults.

Frailty increases in severity with age and is prevalent in older adults [20–22]. The frailty assessment is a practical tool for risk stratification in older adults. It can be used as the basis for the preoperative assessment of older adults [23], evaluating their organ function status, predicting tolerance to surgery, and evaluating the risk of postoperative complications. Accurate assessment of frailty in older peri-operative patients can better guide doctors in controlling the safety of peri-operative patients. Frailty is associated with poor surgical outcomes and prognosis [24]. The risks of surgery and perioperative complications are increased in older patients with frailty [25, 26].

Because short and simple instruments are most feasible in clinical practice, several quick screening tools have been developed and validated [27], including the FRAIL scale. The FRAIL scale is based on self-reported fatigue, mobility, strength, weight loss and the total number of comorbidities and is suitable for screening frail older adults. Frailty is an independent risk factor associated with a high incidence of postoperative adverse events. However, it is difficult to assess the weight loss item of the FRAIL scale because most people do not measure weight in their daily lives. Hence, frail persons cannot determine whether their body weight will fall by 5% in 1 year.

Therefore, the FRAIL scale needs to be improved to be more suitable for preoperative evaluation in clinical practice. Considering that falls are closely related to functional and physical status and that the TUGT is simple, accurate, and easy to perform, we replaced weight loss with the TUGT as a means of preoperative assessment of frailty, with the remaining four items unchanged, and named it the FRAIT Scale. This helped to evaluate the feasibility of the FRAIT scale in predicting the clinical outcomes of hospitalized patients undergoing hepatectomy.

To compare the two scales, this study used perioperative outcomes as the main items, which included factors such as operation duration, bleeding, complications, and death. For the evaluation of complications, the authors’ previous research confirmed that the Clavien‒Dindo classification is an effective method for evaluating complications after hepatectomy [12]. The Clavien–Dindo classification was used to classify the complications in this study. The results showed that the two scales had similar efficacy in predicting blood loss, complications, and death. When predicting operation duration, in addition to increasing the AUC for the FRAIT scale, the new modified scale can predict operation duration. Therefore, the predictive effect of the FRAIT scale was stronger than that of the FRAIL scale. In this study, the TUGT was used instead of weight loss to form a new scale, which proved to be better than the previous scale in predicting operation duration. This is because the TUGT can better reflect body frailty than weight loss. The TUGT reflects the body’s autonomous behavior ability, including cognitive state, muscle strength, balance ability, and other aspects. It is a comprehensive reflection of the body’s state, while weight loss is not necessarily a sign of frailty because in some cases, avoiding bad eating habits and performing aerobic exercise can reduce body weight while improving physical quality.

Not only are the main factors affecting operation duration the type of operation and the complexity of the primary lesion, but the operation duration is also closely related to the effect of anesthesia. Frailty is related to patients’ underlying diseases and poor functional reserve of major organs, such as the heart, lung, liver, and kidney, which increases the risk of anesthesia. Thus, frailty can indirectly affect operation duration by affecting the duration of anesthesia. To maintain the safety of the entire anesthesia process, the anesthesiologist needs to spend more time, and the surgeon should be gentler and more careful during the surgery, which also increases the operation duration.

Frailty seems to be a more common condition in patients with malignant diseases; however, there was no significant difference between malignant and benign diseases in this study. The specific reasons are as follows: most of the benign lesions in this group were hepatolithiasis. Because hepatolithiasis often causes infections and jaundice, the patient’s body often becomes weak owing to these complications [28]. Therefore, in this group of patients, there was no significant difference in the FRAIL/FRAIT scores between the patients with malignant and benign lesions.

In this study, all 251 cases were assessed using the FRAIT and FRAIL scales, and the differences in the scores of these patients and the value of these scales in predicting peri-operative outcomes were compared. The aim of this study was to compare the assessment efficacy of the FRAIT and FRAIL scales. We also verify whether the new FRAIT scale is effective. The content of this study is the phased research results obtained at present, which has preliminarily confirmed the relationship between the FRAIL and FRAIT scales. In future studies, the new FRAIT scale will be validated in all aspects (including short-term outcome and long-term prognosis). Furthermore, we will verify the predictive effect of the scale on surgical outcomes apart from liver surgery with a larger sample. We have previously established a liver surgery risk assessment system [29]. Subsequently, we will perform a regression analysis with FRAIT and other peri-operative factors to improve the previously established liver surgery risk assessment system.

## Conclusions

The frailty status assessed using the FRAIL/FRAIT scales is related to the peri-operative outcomes of hepatectomy. They can be used to assess the frailty status of older patients undergoing hepatectomy before surgery and predict their peri-operative outcomes. The predictive effect of the FRAIL/FRAIT scale scores was stronger than that of their grades. The items of the new FRAIT scale are easier to assess than those of the FRAIL scale, and the FRAIT scale is better than the FRAIL scale in predicting the perioperative outcomes of hepatectomy.

Because of rapid growth of the aging population, our team has adopted a peri-operative frailty assessment as part of routine clinical work. Frailty assessment can better predict perioperative complications, mortality, and other adverse outcomes, which can guide the choice for both doctors and patients and improve patient prognosis. The new FRAIT scale was used to evaluate patient frailty before hepatectomy. Because of its simplicity and easy application, it can be an early warning tool for high-risk older patients undergoing hepatectomy and a reference for peri-operative management.

## Data Availability

The datasets used and analyzed during the current study are available from the corresponding author upon reasonable request.
