# Correction: Efficacy of a modified FRAIL scale in predicting the peri-operative outcome of hepatectomy in older adults (aged ≥ 75 years): a model development study

**DOI:** 10.1186/s12877-024-04886-6

**Published:** 2024-03-27

**Authors:** Lining Xu, Weiyu Wang, Yingying Xu, Bo Yang

**Affiliations:** 1 Department of General Surgery, The Second Medical Center & National Clinical Research Center for Geriatric Diseases, Chinese PLA General Hospital, 100853 Beijing, China; 2 Institute of Hepatobiliary Diseases of Wuhan University, National Quality Control Center for Donated Organ Procurement, Hubei Key Laboratory of Medical Technology on Transplantation, Zhongnan Hospital of Wuhan University, Transplant Center of Wuhan University, 430071 Wuhan, China; 3 Department of Internal Medicine, Henan Cancer Hospital, 450003 Zhengzhou, China; 4 Department of Radiology, Affiliated Union Hospital, Tongji Medical College, Huazhong University of Science and Technology, 430022 Wuhan, China

**Correction: BMC Geriatrics (2023) 23:770**

10.1186/s12877-023-04488-8

After publication of this article, the authors reported that in Tables 2 and 3, the rows starting with ‘Age (years)’ were incorrect and should be removed.

The original article has been corrected.

